# Assessment of Functional Pain Score by Comparing to Traditional Pain Scores

**DOI:** 10.7759/cureus.16847

**Published:** 2021-08-03

**Authors:** Adeolu Adeboye, Rachel Hart, Sri HarshaVardhan Senapathi, Naaila Ali, Lee Holman, Harris W Thomas

**Affiliations:** 1 Surgery, Guthrie Clinic and Robert Packer Hospital, Sayre, USA; 2 Surgery/Trauma and Critical Care, Guthrie Clinic and Robert Packer Hospital, Sayre, USA; 3 General Surgery, Guthrie Clinic and Robert Packer Hospital, Sayre, USA; 4 Medicine, Lake Erie College of Osteopathic Medicine (LECOM), Erie, USA; 5 Anesthesiology/Pain Management, Guthrie Clinic and Robert Packer Hospital, Sayre, USA

**Keywords:** pain rating, pain assessment specialists, postoperative pain, function, chronic and acute pain management

## Abstract

Background: Pain assessments, such as the Numerical Pain Scale (NPS) and Wong-Baker FACEs (FACEs), offer methods to quantify pain with simplistic descriptions on a scale of 0-10 or facial expressions. These tools have limitations and deliver insufficient information to the provider developing a pain management plan. A new Functional Pain Scale (FPS) assesses other scopes of pain, including the loss of function in activities of daily living, sleep habits, and communication. Although NPS and FACEs are traditionally used in clinical practice, FPS provides a functional assessment to help patients self-report their pain to their providers.

Aim: Our study attempts to show a comparative data analysis of the FPS to NPS and FACEs. The purpose of our study is not to demonstrate FPS's superiority over NPS and FACEs but to fill the gaps of information necessary to communicate the type of pain a patient has to their provider. Due to its descriptive nature and clear scores, FPS should be implemented within electronic medical records (EMR) to help providers assess patients’ pain and evaluate the efficacy of interventions selected based on that pain.

Design: A prospective, observational, single-center, cohort study was performed, with simultaneously administered surveys to compare pains scores on a new FPS to the common NPS and FACEs. The target sample was postoperative orthopedic patients above 18 years of age who can read and speak English. Patients were surveyed on all three pain scales: FPS, NPS, and FACEs and were asked to rate their pain perioperatively after their respective orthopedic procedures.

Methods: Spearman correlation method was used to test for correlation between the three pain scales and Wilcoxon rank-sum test was used to compare means between FPS and NPS.

Results: FPS has a strong correlation with FACEs (r = 0.647, p<0.05) and with NPS (r = 0.634, p<0.05). There is a significant difference in mean scores between FPS and NPS.

Conclusion and study implications: The most reliable marker of pain is patient self-reporting. In routine assessment, because pain is one-dimensional, we as providers need to better define the range of 0-10. This can only be done via an algorithm regarding which functions are lost as pain intensities increase. FPS fits those requirements by offering suitable descriptions with each pain score. The implications of the study include a chance to remedy the opioid crisis that plagues healthcare. We need tools that assess and educate patients about their pain level and appropriately convey that information to providers. Furthermore, this study is a chance for innovative tools to be implemented to better change healthcare practice. If FPS gains traction, it can improve pain communication between patients and providers.

## Introduction

Pain is considered the fifth vital sign for clinical practice [1]. The provider's responsibility is to help patients discuss pain and its intensity with clarity and ease. Pain is defined as "an unpleasant sensory and emotional experience associated with actual and potential tissue damage" [2]. Self-reporting of pain is considered the most reliable way for a patient to communicate their pain experience [3]. Common self-reporting tools in hospitals include the Numerical Pain Scale (NPS) and the Wong-Baker FACEs (FACEs) scale. NPS was implemented in clinical practice due to its rapidity both verbally and in writing [4]. The design of NPS was simplistic, addressing a range of 0 to 10 with 0 being "no pain" and 10 being "worst pain." Due to its convenience, it is common for hospitals to measure pain using the 0 to 10 NPS scale. However, a key drawback of NPS is that the difference between pain scores (such as the difference between 2 and 4 or the difference between 6 and 8) may not be thoroughly comparable in scaling pain intensity. Another limitation of NPS is that the terms "least pain" or "worst pain" depend on a patient’s perspective and prior pain experience, and vary from patient to patient. Men in comparison to women may vary in how they interpret "worst pain experienced," thus altering uniformity in the definitions of 0 to 10 [5].^ ^FACEs approach pain with a more visual representation using number ranges in the context of facial expressions to convey a patient's pain experience. Initially, FACEs were developed for pediatric populations to communicate to providers, but its success expanded the scope of the scale to include adults. Fundamentally, FACEs were considered an adequate scale; however, it is weak in lack of standardization in translating a pain experience. There is an extensive debate about what constitutes a happy face versus a sad face. Patients of various cultural backgrounds may find a facial shape, features, and spectrums of pain (such as furrowing of the forehead, the elevation of eyebrows, or mouth opening) as controversial components of what defines pain [6]. Another issue with the pain scale is difficulty in communicating improvement in pain in FACEs scores when each face constitutes a number range that is a multiple of 2. If a provider were trying to detect if an analgesic showed an improvement, a score of 7 would be the same as 8 when corresponding to the same face on FACEs.

Although the NPS and FACEs evaluate pain intensity sufficiently and convey information for providers to control their pain, these instruments are limited in their standardization of definitions for what corresponds to each numerical value. There should be defined changes between each score as it increases or decreases with intensity. That is where the new FPS displays clinical advantage. Not only does the FPS have a range of 0 to 10, but it also measures how pain affects function. This scale function covers activities of daily living, sleeping habits, and communication. As intensity increases, more critical elements of function are affected, with the highest pain leaving the patient debilitated. Tools that integrate functional impairment with a parallel number offer a structure for the patient, eliminate interpretation, and instill consistency in pain scoring (table in the Appendix). Interestingly, the trend in past studies has shown that patients routinely rank their pain at the highest standard to assure they are receiving adequate pain medications [4]. These measures can be easily eliminated with the use of FPS. Patients will reconsider marking a high score if it lacks equivalence to their functional components [7].

Primarily, this study aims not to demonstrate the superiority of FPS over NPS and FACEs but to fill the gaps of information necessary to communicate to their provider the type of pain a patient has. Our study attempts to show a comparative data analysis of FPS to NPS and FACEs. Due to its descriptive nature and analogous scores, the new FPS should be implemented within the electronic medical records (EMR) to help providers assess pain and evaluate efficacy of interventions selected based on that functional pain score.

## Materials and methods

This study was completed over three months at the Guthrie Robert Packer Hospital with a participant population of 49. The study was an observational cohort study. The study evaluation was completed on postoperative day (POD) #1 on orthopedic patients required to undergo hip or knee replacements. The patients above 18 years of age who can read and speak English were included. These patients were selected from an orthopedic field due to the uniformity of pain. Some procedures did not necessarily need aggressive pain management measures. Once the selection criteria were met, the patients were selected before surgery, and invitations were extended during preadmission testing.

Data collection consisted of administering FPS, NPS, and FACEs scales to survey patients after their procedure. Surveys were administered simultaneously to assess convergent validity, analysis representing the degree to which an evaluation agrees with other valid measures of a similar idea. The FPS scale provides a score based on the description of pain. The score ranges from 0 to 10, with 0 accounting for no pain and 10 being immobilizing pain. Each number corresponds to a thorough description of keywords meant to describe pain, including minimal (1), mild (2), uncomfortable (3), moderate (4), distracting (5), distressing (6), unmanageable (7), intense (8), and severe (9). The numbered descriptions focus on activities of daily living (ADL), sleep habits, and communicability with others (table in the Appendix).

NPS and FACEs were selected for comparison since they are commonly used in the clinical setting and have a similar design for pain assessment. The reliability and validity of NPS and FACEs have been shown over many patient populations [4]. In NPS, the scoring is in the range of 0 to 10 with zero representing "no pain at all" and 10 representing the "worst pain ever experienced" [8]. Patients are asked to indicate what number best corresponds to their current intensity of pain. FACEs scale utilizes six ordinal, color-coded faces with globally recognized facial expressions. These expressions correspond to scores that are multiples of two. For our analysis, the FACEs were scored as 0, 2, 4, 6, 8, and 10.

For statistical analysis of the three pain scales, a Spearman correlation test was used to test for correlation between the existing pain scales and the FPS. Wilcoxon's paired t-test was used to measure if there is a difference in mean pain scores on the FPS and NPS. The overall data analysis was performed on R version 3.5.1 (R Foundation for Statistical Computing, Vienna, Austria).

## Results

Patient characteristics and their respective pain score summaries are shown in Table 1. The total sample size consisted of 49 patients. The mean age of the patients was 65.8 years with SD of 7.47 years. Of the total patients, 28 patients (57.1%) were female. The median (range) pain scores on the FPS, NPS, and FACEs were 4 (1,9), 4 (1,8), and 4 (2,8). The mean (standard deviation) pain scores for FPS, NPS, and FACEs were 3.65 (1.75), 4.53 (2.12), and 4.78 (2.23). The mean pain score indicated on the Numerical Pain Scale is significantly higher than the score from the Function Pain Scale with a p-value < 005. In terms of procedure breakdown, most of the patients (32.7%) had an arthroplasty total knee persona.

**Table 1:** Patient characteristics and pain score summary

Patient Characteristics and Pain Score Summary	Overall
	(N=49)
Age	
Mean (SD)	65.8 (7.47)
Median (min, max)	67.0 (45.0, 78.0)
Gender	
Female	28 (57.1%)
Male	21 (42.9%)
Functional Pain Score	
Mean (SD)	3.65 (1.75)
Median (min, max)	4.00 (1.00, 9.00)
Numerical Pain Score	
Mean (SD)	4.53 (2.12)
Median (min, max)	4.00 (1.00, 8.00)
FACEs	
Mean (SD)	4.78 (2.23)
Median (min, max)	4.00 (2.00, 8.00)
Procedure	
Arthroplasty Total Knee, Persona Bilateral	1 (2.0%)
Arthroplasty Total Hip, Continuum Taperloc	8 (16.3%)
Arthroplasty Total Hip, Magnum Taperloc	1 (2.0%)
Arthroplasty Total Knee, Smith and Nephew	1 (2.0%)
Arthroplasty Total Knee, Persona	16 (32.7%)
Arthroplasty Total Hip, Stryker	2 (4.1%)
Hemiarthroplasty Knee, Oxford	1 (2.0%)
Hemiarthroplasty Knee	2 (4.1%)
Makoplasty Total Hip	2 (4.1%)
Makoplasty Total Knee	15 (30.6%)

**Figure 1:**
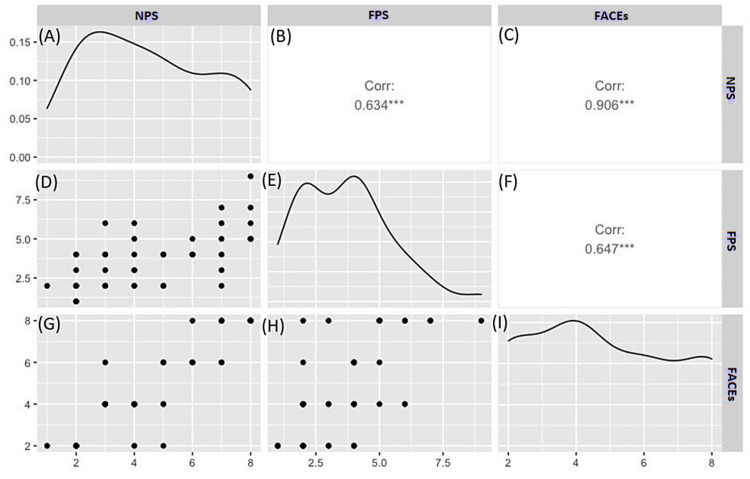
Correlation between the three pain scales (NPS, FPS, FACEs) and their distributions (A) Histogram of raw data of NPS; (B) correlation graph of FPS vs. NPS; (C) correlation of FACEs vs. NPS; (D) scatter plot of NPS vs. FPS; (E) histogram of raw data of FPS; (F) correlation of FACEs vs. FPS; (G) scatter plot of NPS vs. FACEs; (H) scatter plot of FPS vs. FACEs; (I) histogram of raw data of FACEs. NPS: Numerical Pain Scale; FPS: Functional Pain Scale; FACEs: Wong-Baker FACEs

Figures 1A-1I illustrate a correlation between the three pain scales and their distributions. Raw data are shown as a histogram for FPS, NPS, and FACEs (Figures 1A, 1E, 1I). Scatter plots depicting FPS, NPS, and FACEs comparisons are shown in Figures 1D, 1G, 1H. Scatter plots illustrate composite correlation coefficients shown in Figures 1B, 1C, 1F. FPS vs. NPS had a positive correlation coefficient of 0.634 (Figure 1B). FPS vs. FACEs had a similar positive correlation coefficient of 0.647 (Figure 1F). FACEs vs. NPS had the highest correlation coefficient of 0.906 (Figure 1C). Overall, in Figure 1, the FPS scores are significantly correlated with the scores on NPS and FACEs with a p-value < 0.05.

**Figure 2:**
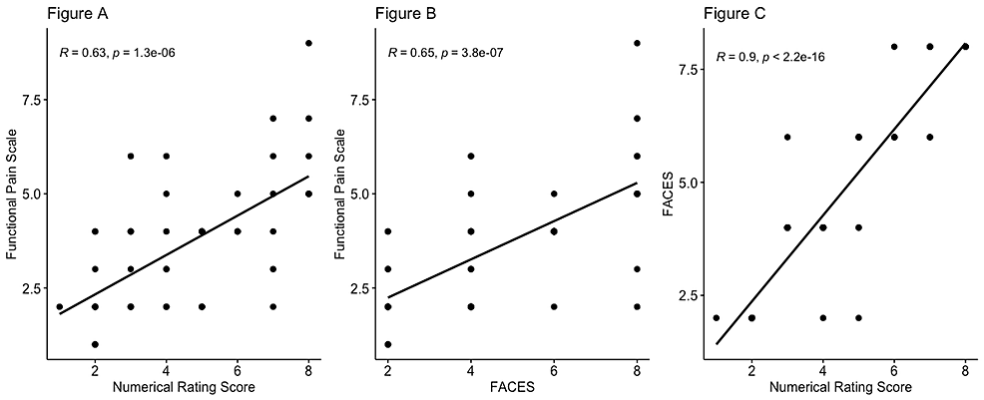
Comparison of FPS, NPS, and Wong-Baker FACEs scales (A) Comparison between FPS and NPS; (B) comparison between FPS and Wong-Baker FACEs; (C) comparison between Wong-Baker FACEs and NPS NPS: Numerical Pain Scale; FPS: Functional Pain Scale; FACEs: Wong-Baker FACEs

Figures 2A-2C illustrate the line of best fit for comparisons made between FS, NPS, and FACEs. All three pain scales have positive lines of best fits for each scatter plot. 

## Discussion

The goal of our study was to evaluate how comparable the FPS is to NPS and FACEs, and we observed a significant correlation between the FPS and the current pain scales. While NPS and FACEs have been used in the clinical setting over many years, we wanted to demonstrate that FPS is an important descriptive and reputable pain scale. Through convergent validity, we found that FPS has a positive correlation coefficient with NPS of 0.634 (Figure 1B). This is further emphasized with the moderate positive line of best fit of NPS vs. FPS (Figure 1A). The likelihood that FPS and NPS were similarly scored in the patient population is due to the similarity of the surveys in definitions of keywords. FPS refers to 10 as "unable to move or talk due to intensity of pain," and it is a severe range order. Similarly, NPS refers to 10 as "worst pain experienced in life." Both questionnaires utilize similar vocabulary, showing why scores were correlated [1]. In addition, FPS and FACEs had a higher positively correlated ratio of 0.647 (Figure 1F). This is again emphasized with a moderate positive line of best fit of FACEs vs. FPS (Figure 1B). This is expected since the facial expression in FACEs has similarity to the description found in FPS. For example, ranking 7 to 8 on FACEs corresponding to an unhappy face would have a similar rating on FPS with the pain score described as "unmanageable" or "intense" and a patient "can't concentrate" and is "unable to sleep" (table in the Appendix). It is important to note that the highest positive correlation coefficient lies between NPS vs. FACEs at 0.906 (Figures 1C, 2C). We suggest that this correlation is due to the longevity of NPS and FACEs being used and refined through the years to improve the validity of the scales. However, aside from scoring the pain, NPS and FACEs do not convey any qualitative information about how the patient is functioning with the pain. While there is a significant correlation, we found the mean FPS score to be significantly lower than the NPS and FACEs. The median pain scores on the FPS, NPS, and FACES were 4 (1,9), 4 (1,8), and 4 (2,8). The mean pain scores for FPS, NPS, and FACEs were 3.65 (SD 1.75), 4.53 (SD 2.12), and 4.78 (SD 2.23) with a significant difference between the three pain scales (Table 1). We believe the lower pain score on FPS is due to a predefined description of each pain point and a standardized definition of each pain point.

The FPS showed some advantages and uncovered some limitations of the current scales. NPS provides only a value and FACEs offer a nondescript range. The main drawback in FACEs was the misinterpretation of ordinal facial expressions due to varied perceptions of happy and sad [6]. We believe the FPS to be an improved scale for patients of all backgrounds. We want providers to have the most information when treating pain. FPS has a renowned range of 0-10, and it measures how pain affects function. The algorithm devised shows the effects on sleep, ability to complete ADLs, and impact on communicability. Providers need a tool that can help the healthcare team do an objective assessment without losing realistic recovery goals.

There are limitations to the FPS that we observed during our study. The main weakness was the lack of assessment for the cognitive ability of our patients. Many of the words in the FPS require specific education or level of understanding. Particularly older adults have alterations in cognitive, sensory-perceptual, and motor abilities, which leave them incapable of communicating or quantifying their pain. Examples of this consist of presbycusis, dementia, delirium, and non-English speaking [3]. The words in FPS may need to be explained to some patients and cognitive functions should be assessed prior. Another limitation is the small sample size and the single-center study conducted in orthopedic patients. To assess generalizability, future studies should extend the use of FPS to other patient populations rather than just orthopedic procedures. While orthopedic patients offer an excellent sample for pain assessment, the study should be extended to areas such as obstetrics and gynecology (OB-GYN), plastics, general surgery, vascular, and the inpatient setting.

A major issue ailing healthcare is the improper selection of pain medications. Patients consistently rank their pain at the highest standard to assure they are receiving satisfactory analgesics [7]. Patients can no longer continue this trend with the recent opioid crisis of 2018. When the Department of Enforcement Agency and Department of Justice pushed pharmaceuticals to cut production of opioids, they did this to battle the national shortages of narcotics [9]. We can no longer squander opioids but make sure they are appropriated wisely. Healthcare providers need an informative tool that fairly prescribes pain medications. FPS meets those expectations by offering a quantitative measure as well as in-depth definitions for each pain intensity's loss of function. In one report, patients attempted to report their pain as immobilizing (10 out of 10), but these patients exhibited functional or stable conditions and some had signs of "narcotic associated sedation." When a follow-up interview was performed, the patients stated they did not understand the numbers on the pain scale or feared they would not be treated if they did not report 10 [10]. Because of FPS checkpoints with descript losses of function, providers were prevented from overtreating the patient. Essentially, FPS offered a more effective, consistent, and straightforward form of communication.

Taken together, the traditional pain scores (NPS and FACEs) have been highly subjective, inconsistent, and poorly defined [10]. What we need is a process where the providers' perception of the pain score meaning is the same as the patient's opinion. We want patients to feel their self-report of pain has value and that as providers, we are fully aware of their experience. If we can closely match both ends of expectations, then we can effectively meet pain management goals as well as patient satisfaction.

## Conclusions

Pain is a critical vital sign of a patient's overall profile. The most reliable marker of pain is to self-report. In routine assessment, providers need to better define the range of 0-10 so that patient self-reports are accurate. This can only be done via an algorithm of specific pain intensities associated with loss of functions. The FPS fits this requirement by offering adequate descriptions of the ability to function with each pain score. This study of orthopedic patients shows that the FPS has convergent validity with the other pain scales (NPS and FACEs). More research is indeed needed to gain traction for FPS, but through this small study, clinical practices are now aware of a new assessment that better approaches the quantification of pain and better direction for treatment.

## Appendices

**Table 2:** Functional Pain Scale - descriptive words and definitions ADL: activities of daily living

Descriptor	Definition
No Pain (0)	No pain
Minimal (1)	Hardly noticeable/no impact on ADL’s/sleep not affected and able to use passive distraction for comfort. Mild range order
Mild (2)	Noticeable when not distracted/no impact on ADL’s/sleep only slightly affected and able to use both passive and active distraction for comfort. Mild range order
Uncomfortable (3)	Pain is present but can complete all ADL’s/sleep is slightly affected and passive distraction only gives marginal relief. Mild range order
Moderate (4)	Constantly aware of pain but can complete ADLs with modification/sleep marginally affected at times/passive distraction is of no use, but active distraction gives some relief. Moderate range order
Distracting (5)	Aware of pain/able to complete some ADL’s but limited by pain/sleep is affected and active distractions are only slightly useful. Moderate range order
Distressing (6)	Pain is present/unable to complete most ADLs limited by pain/sleep is difficult and active distraction is only marginal. Moderate range order
Unmanageable (7)	Pain interferes with normal ADL’s/nothing seems to help/sleep is very difficult/active distractions are very difficult to concentrate on. Severe range order
Intense (8)	Cannot complete any ADLs without much assistance/cannot concentrate/conversation is difficult/unable to sleep and unable to use distraction. Severe range order
Severe (9)	Cannot do any ADL’s even with assistance can barely talk/unable to sleep and unable to use distraction. Severe range order
Immobilizing (10)	Unable to move or talk due to intensity of pain/unable to sleep and unable to use distraction. Severe range order
